# T-LAK cell-originated protein kinase (TOPK): an emerging target for cancer-specific therapeutics

**DOI:** 10.1038/s41419-018-1131-7

**Published:** 2018-10-24

**Authors:** Katharine J. Herbert, Thomas M. Ashton, Remko Prevo, Giacomo Pirovano, Geoff S. Higgins

**Affiliations:** 1 CRUK/MRC Oxford Institute for Radiation Oncology, University of Oxford, Old Road Campus Research Building, Roosevelt Drive, Oxford, OX3 7DQ UK; 2 Department of Radiology, Memorial Sloan-Kettering Cancer Center, New York, NY USA

## Abstract

‘Targeted’ or ‘biological’ cancer treatments rely on differential gene expression between normal tissue and cancer, and genetic changes that render tumour cells especially sensitive to the agent being applied. Problems exist with the application of many agents as a result of damage to local tissues, tumour evolution and treatment resistance, or through systemic toxicity. Hence, there is a therapeutic need to uncover specific clinical targets which enhance the efficacy of cancer treatment whilst minimising the risk to healthy tissues. T-LAK cell-originated protein kinase (TOPK) is a MAPKK-like kinase which plays a role in cell cycle regulation and mitotic progression. As a consequence, TOPK expression is minimal in differentiated cells, although its overexpression is a pathophysiological feature of many tumours. Hence, TOPK has garnered interest as a cancer-specific biomarker and biochemical target with the potential to enhance cancer therapy whilst causing minimal harm to normal tissues. Small molecule inhibitors of TOPK have produced encouraging results as a stand-alone treatment in vitro and in vivo, and are expected to advance into clinical trials in the near future. In this review, we present the current literature pertaining to TOPK as a potential clinical target and describe the progress made in uncovering its role in tumour development. Firstly, we describe the functional role of TOPK as a pro-oncogenic kinase, followed by a discussion of its potential as a target for the treatment of cancers with high-TOPK expression. Next, we provide an overview of the current preclinical progress in TOPK inhibitor discovery and development, with respect to future adaptation for clinical use.

## Facts

- TOPK activity facilitates cell cycle control and mitotic progression.
- Dysregulated TOPK expression potentiates cancer development and dissemination.
- High-TOPK expression is tumour-specific and is associated with poor clinical outcomes.
- TOPK inhibitors have anti-cancer potential when combined with chemo- or radiotherapy.

## Open questions

- What process contributes to dysregulation of TOPK expression during cancer progression?
- Are there parallels between the function of TOPK in embryonic stem cells and its behaviour in cancer stem cells?
- How does TOPK suppression contribute to the cancer-specific potency of DNA damaging therapies?
- Can TOPK inhibition function as a low risk alternative to MEK or CDK inhibition in the clinical treatment of TOPK overexpressing cancers?

## Introduction

T-LAK cell-originated protein kinase (TOPK), also known as PDZ-binding kinase (PBK), is a dual specificity serine/threonine kinase with sequence homology lying between the phylogenetic branches for MEK1/2 and MEK7, but with greater functional similarity to the p38-activating MEKs, MKK3/6^1^. As a member of the dual specificity family of kinases, it was anticipated that TOPK would play a role in activation of JNK, ERK and Akt. Early studies suggested that TOPK facilitates transformation by upregulating and activating ERK2 through an increase in AP-1 transcriptional activity^2,3^. Indeed, a positive feedback loop between TOPK and ERK2 was found to amplify kinase signalling in HCT116 colon cancer cells. In this cell line, TOPK-mediated pathway activation was independent of B-Raf or C-Raf, leading the authors to conclude that a TOPK/ERK2 feedback loop may be contributing to transformation potential by bypassing the negative feedback loop regulating the Raf/MEK/ERK pathway^3^. This was later confirmed when TOPK was shown to activate ERK independently of MEK1/2 as part of the EGFR signalling pathway, and to contribute to MEK inhibitor resistance in MCF7 cells^4^. As a novel regulator of downstream Raf/MEK/ERK pathway activity, TOPK, therefore, provides an attractive potential target for chemotherapeutic inhibitor development.

## Localisation, tissue distribution and function

TOPK expression is largely confined to tissues with high levels of proliferation. Its interest as an oncogenic target lies in the differential expression of TOPK in multiple tumour types relative to their normal tissue counterparts^5–8^. Despite this, TOPK knockout does not confer embryonic lethality in mouse models, with the only phenotype reported being an impaired inflammatory response to UV light exposure in the skin^9^. TOPK is expressed in both the nucleus and cytoplasm, however, some reports indicate that nuclear expression is exclusive to tumour cells and to those undergoing mitosis^6^, whilst others do not detect any difference in localisation^10^. TOPK mRNA is detected in several tissue types; and is most abundant in human tissues derived from testis, placenta and thymus^1,11,12^. Of these, the highest signal is measured in testicular tissue, with expression in this organ confined exclusively to spermatogenic germ cells in situ^13^. In brain tissue, TOPK-expressing cells arise from GFAP-positive neural progenitor cells in the adult sub-ependymal zone as well as in the external granule cell layer of the postnatal cerebellum^14^. Its expression is exclusive to proliferating and multipotent neurogenic precursors, and it is suppressed in mature or quiescent neurons.

**Fig. 1:**
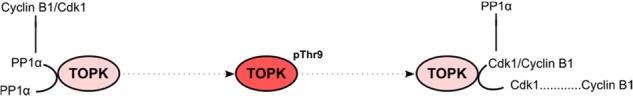
**TOPK activity and expression rises at the G2/M border to facilitate mitotic entry.** Activation of TOPK is initiated by Cdk1-mediated dissociation of protein phosphatase 1 alpha (PP1α). TOPK activity is instrumental in the stabilisation of the Cdk1/Cyclin B complex between prophase and metaphase, which promotes spindle formation and correct chromosomal alignment. Rapid dephosphorylation of TOPK at the metaphase/anaphase border (mid-mitotic checkpoint) releases TOPK from Cdk1, enabling proteolytic degradation of Cyclin B by the anaphase promoting complex (APC). A drop in Cyclin B levels enables chromosomal migration to spindle poles and progression into anaphase

**Fig. 2:**
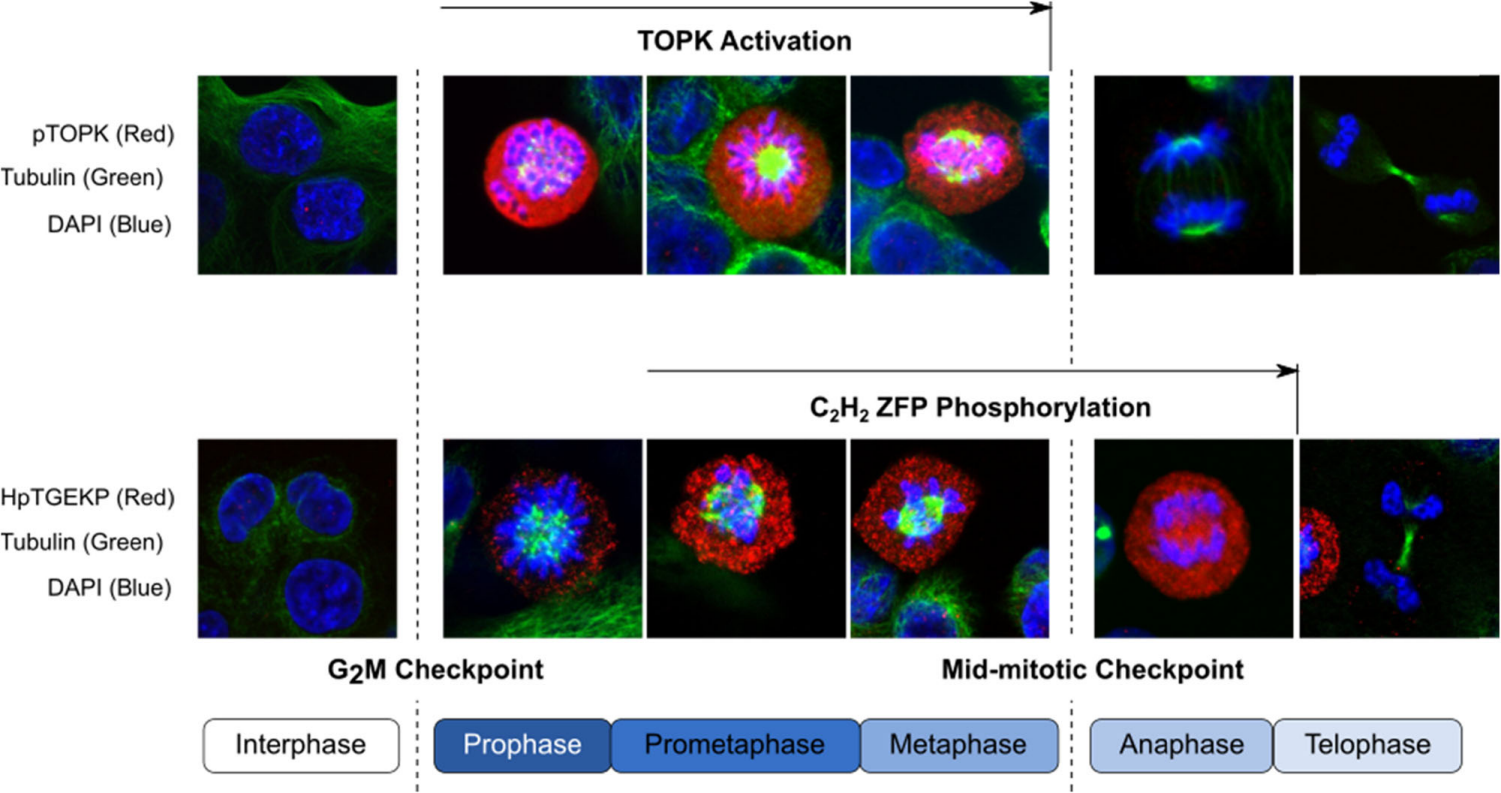
**TOPK activity facilitates mitotic progression.** Fluorescent images depict the association between mitotic stage and TOPK activity. Upper row is stained for TOPK phosphorylated at its Thr9 residue (red). Lower row is stained for proteins bearing the phosphorylated form of the C2H2 zinc finger consensus linker sequence motif, HpTGEKP (red). Phosphorylation at this motif coordinates the dissociation of C2H2 zinc finger proteins (ZFP) from condensed chromatin, enabling mitotic progression^56^. TOPK has been identified as the primary kinase responsible for phosphorylation of HpTGEKP bearing proteins during mitosis^18^. Tubulin is counterstained green, and DNA is identified by DAPI staining (blue)

TOPK expression and phosphorylation consistently increases as proliferating cells enter mitosis^13^; and this process is regulated by Cdk1 in a Cyclin B1-dependent manner^12,15^. Although TOPK expression is closely associated with Cdk1 during the cell cycle^16^, the relationship between Cdk1 and regulation of TOPK phosphorylation is instead mediated via inactivation of protein phosphatase 1α (PP1α) through the Cyclin B1/Cdk1 complex^17^. Inactivation of PP1α causes TOPK to become phosphorylated at Thr9, promoting TOPK activation (Fig. 1). TOPK activity is primed to peak on entry into prophase, when it binds to and phosphorylates mitotic C_2_H_2_ zinc finger proteins via its PDZ-binding domain^18^, and contributes to chromatin condensation by facilitating phosphorylation of histone H3 at Ser10^19^. Thus TOPK activity and expression contribute to the release of proliferating cells from mid-mitotic checkpoint control and maintain a timely exit from mitosis (Fig. 2).

## Preclinical rationale: TOPK overexpression in cancer

High expression of TOPK has been linked to tumour aggressiveness, invasion and metastatic spread. Indeed, testing of clinical samples has revealed that a significant relationship exists between TOPK expression and poor prognosis in numerous cancers (Table 1). TOPK overexpression in cancer appears to be facilitated by suppression of microRNA-mediated regulatory control. Restoration of miR-216b-3p expression in lung cancer cell lines is sufficient to inhibit proliferation, enhance apoptotic cell death and overcome TOPK-associated down-regulation of p53 and p21^20^.

**Table 1:** TOPK expression is a prognostic marker for cancer

Site	Cancer type	Methodology	Findings	References
Prostate	Neuroendocrine carcinoma, acinar adenocarcinoma	Transcriptome profiling, IHC	One of the top 10 genes overexpressed in small cell carcinoma	^57^
	Adenocarcinoma	IHC, qRT-PCR	Upregulation associated with age, Gleason score, clinicopathological stage, metastatic spread, survival and PSA failure. Predictor for biochemical recurrence-free survival	^10^
	Prostatic adenocarcinoma	IHC, immunoblot, microarray	Expression correlates with lack of tissue differentiation, disease aggression and metastatic spread	^6^
	Prostatic adenocarcinoma	Custom qRT-PCR Microarray	Cancer/testis antigen for prostate cancer, expression correlates with Gleason score but not age, stage, or preoperative PSA	^58^
	Prostatic adenocarcinoma	IHC	Expression correlates with stage > T2c and Gleason score ≥ 8, PSA > 20 ng/ml	^59^
Liver	Hepatocellular carcinoma, cholangiocarcinoma	IHC	High expression in cholangiocarcinoma, lower expression in hepatocellular carcinoma. Low expression associated with poor prognosis in CCA	^60^
	Hepatocellular carcinoma	qRT-PCR; immunoblot	Upregulation in **all** HCC cases	^61^
Head and neck	Oral squamous cell carcinoma	IHC	Low expression associated with poor prognosis in young patients, high expression in older patients associated with poorly differentiated tumours, smokers, and late-stage disease.	^62^
Ovarian	Epithelial ovarian cancer	IHC, Immunoblot, qRT-PCR	High expression associated with poor progression-free survival and overall survival in early-stage cancer; Transactivation in EOC, less in borderline malignancy tumours	^44^
Brain	Glioblastoma multiforme	Immunoblot, IHC qRT-PCR	Upregulated in all GBM samples	^7^
Lung	Adenocarcinoma	IHC	High expression associated with poorly differentiated tumours, metastatic spread, high-TNM stage and reduced overall survival. Patients with combination of high TOPK and mutp53 had lowest prognosis	^63^
Haematological	Primary AML	qRT-PCR, immunoblotting	TOPK detected in 9/12 samples; 3/3 ALL samples and in plasmocytoma and blastic type mantle cell lymphoma. Weak expression in peripheral blood stem cells	^64^
Gastric	Gastric carcinoma	IHC, immunoblotting	Expression independently correlated with poor outcome	^65^
Oesophageal	Oesophageal squamous cell carcinoma	qRT-PCR, IHC, immunoblotting	Expression independently correlated with poor outcome	^66^
Colorectal	Colorectal carcinoma	IHC tissue microarray	No relationship between VEGFa gene amplification and TOPK expression/gene status	^67^

Expression of TOPK in primary tumours is linked to enhanced tumour aggression, invasion and metastatic spread in a variety of cancers. Multivariate analysis of clinical samples from numerous sites indicates that a significant relationship exists between TOPK expression in tumour tissue and poor prognosis for patients.

Key: *IHC* immunohistochemistry, *PSA* prostate specific antigen, *AML* acute myeloid leukaemia, *ALL* acute lymphocytic leukaemia, *TNM* tumour, node and metastasis, *EOC* epithelial ovarian cancer, *GBM* glioblastoma multiform, *CCA* cholangiocarcinoma, *VEGFa* vascular endothelial growth factor A, *TOPK* T-LAK cell-originated protein kinase.

Development of malignant potential is facilitated by TOPK-mediated responses to growth and migratory signalling. An increase in TOPK expression in JB6 epidermal cells accelerates cell growth and anchorage-independent colony formation in vitro, as well as promoting tumour formation in vivo^3,21^. Conversely, suppression of TOPK in cancer cell lines inhibits in vivo tumour growth^3,21,22^. In addition to its modulating effect on growth and migration, TOPK promotes epithelial–mesenchymal transition and facilitates tumour invasiveness. Anchorage-dependent growth and motility is enhanced by TOPK expression in prostate and breast cancer cell lines^5,6,10,23^, and overexpression of TOPK in metastatic prostate cancer is associated with an increase in the expression of matrix metalloproteinases MMP-2 and -9^6^. These changes were attributed to nuclear localisation of β-Catenin and activation of TCF/LEF signalling at gene promotor regions. Nuclear translocation and activation of β-Catenin signalling targets MMP-7, Cyclin D1, and TCF-1 is also enhanced in hepatocellular carcinoma cell lines with increased TOPK expression, a result supported by clinical data showing correlation between high-TOPK levels and nuclear β-Catenin localisation in tissues from hepatocellular carcinoma patients^21^.

High-throughput screening has raised the possibility of increased TOPK expression in cancer being representative of stemness in tumours, however future investigations are needed to clarify the relationship between TOPK and proliferation in cancer stem cells (CSC). Nonetheless, TOPK was ranked fifth in a screen of glioblastoma and breast cancer cell lines conducted to identify potential cancer stem cell targets^24^, and was identified as a marker for proliferation potential in multipotent stromal cells derived from bone marrow^25^. Inhibition of TOPK has been effective in suppressing growth and survival in CSC subpopulations of small cell lung cancer cell lines, an outcome which is thought to be associated with suppression of FOXM1 activity—a key transcription factor regulating expression of late cell cycle control genes^26^. TOPK expression appears to maintain haematopoietic stem cells in an undifferentiated state, as TOPK inhibition enhances production of platelets and megakaryocytes in vivo, and induces myeloid maturation in AML cell lines by downregulating STAT5 protein expression^27,28^. Hence, the interest in TOPK as a potential biomarker for patient stratification and risk assessment in cancers with high incidence of acquired treatment resistance and/or recurrence.

### Therapeutic targeting of TOPK

TOPK has demonstrated potential as a therapeutic target for suppressing cancer development by overcoming treatment resistance, preventing invasion and metastatic growth, and promoting cell death signalling in oncogenic tissues. At a mechanistic level, this appears to occur through multiple interactions, and in a tumour-specific manner—predominantly due to the influence of TOPK overexpression on cancer cell physiology (Table 2).

**Table 2:** TOPK is a pro-oncogenic kinase with chemotherapeutic potential

Characteristic	Mechanism	References
Tumour dissemination	PRPK phosphorylation (Ser250) by TOPK regulates p53- and Akt-mediated activation of tumour cell migration and invasion.	^29^
Proliferative potential, replicative immortality	TOPK expression is regulated by a negative feedback loop via FLT3 expression and CEBPA phosphorylation.	^28^
Apoptotic resistance	TOPK binds histone H2A, promoting nuclear colocalisation and phosphorylation of γH2AX.	^32^
	TOPK and PRX1 colocalise in the nucleus. TOPK regulates PRX1 peroxidase activity by phosphorylation at Ser32.	^33^
Cell death signalling	TOPK suppresses p53-mediated transcription of pro-apoptotic intermediates in tumour cells.	^28, 36^
	TOPK confers resistance to TRAIL-induced apoptotic cell death via NF-κB mediated transcriptional activity.	^37^
	TOPK directly phosphorylates IκBα at Ser-32 and promotes RelA nuclear translocation. Overexpression enhances NF-κB and cIAP2 transcriptional activity.	^38^
Oxidative damage	TOPK activation protects against cell death by enhancing the Bcl-2/Bax ratio.	^34^
	pAkt and pTOPK colocalise in neural cells following ischaemia, increasing expression of peroxiredoxins-1 and 2, and thioredoxin-1.	^35^
	TOPK suppresses JNK/p38 signal pathway activation during exposure to oxidising conditions.	^22^
DNA damage	TOPK expression in cancer cell lines promotes resolution of chromosomal errors following DNA damage.	^40^
	TOPK expression is regulated by E2F and CREB/ATF-mediated transcription. TOPK directly interacts with p53 and promotes molecular destabilisation.	^39^

Increased TOPK expression in cancer cells promotes the activation of multiple pathways involved in sustained growth and proliferation, evasion of cell death, invasive potential and metastatic growth. Inhibition of TOPK activity restores cellular responses to cell death signalling and replicative control mechanisms, as well as overcoming oncogenic chemoresistance by sensitising cancer cells to DNA damaging agents.

Key: *TOPK* T-LAK cell-originated protein kinase, *FLT3* fms-like tyrosine kinase 3 (CD135), *CEBPA* CCAAT/enhancer-binding protein alpha, *NF-κB* nuclear factor kappa light chain enhancer of activated B cells, *TRAIL* TNF-related apoptosis-inducing ligand, *IκBα* inhibitor of kappa B, *CREB/ATF* cAMP response element binding protein activating transcription factor, *PRPK* p53-related protein kinase, *PRX1* Peroxiredoxin 1.

TOPK promotes tumour dissemination by direct phosphorylation of p53-related protein kinase (PRPK) at its Ser250 residue, which in turn regulates the phosphorylation status of p53 and Akt. Through this mechanism, manipulation of TOPK expression in HCT116 colon cancer xenografts is sufficient to influence migration and metastatic growth in mouse liver tissue^29^. Relative expression of TOPK and its downstream target, PRPK, are also related to metastatic potential in colorectal carcinoma and squamous cell carcinoma^30,31^. Further, TOPK knockout mice are resistant to solar stimulated light-induced skin cancer^31^. For patients with acute myeloid leukaemia (AML), myeloid maturation in *FTL3*-ITD positive AML cells appears to be partially suppressed by a feedback loop between TOPK and the transcription factor responsible for granulocyte differentiation, CEBPA. In CD34 positive myeloblasts, inhibition of TOPK in *FTL3*-ITD positive AML promotes apoptotic cell death and increased myeloid differentiation^28^.

TOPK activation increases during oxidative stress, and provides protection to cells in pro-inflammatory environments when overexpressed. TOPK-mediated phosphorylation of γH2AX and peroxiredoxin 1 significantly suppresses induction of apoptotic signalling cascades in melanoma cell lines, conferring resistance to arsenite-induced and UVB-induced apoptotic cell death^32,33^. Overexpression of TOPK was also found to provide protection against myocardial and neuronal ischaemia-reperfusion injury by enhancing activation of the Akt signalling pathway and by upregulating expression of antioxidative proteins^34,35^. In both these investigations, inhibition of TOPK reduced cell viability and aggravated ischaemia-induced injury in vitro and in vivo.

Combination TOPK targeting in cancer therapy regimens has the potential to enhance treatment efficacy when coupled with DNA damaging agents. Suppression of TOPK activity proportionately increases the apoptotic fraction of tumour cells by upregulating pro-apoptotic p53 transcriptional targets, *NOXA*, *BAK*, *FAS* and *Caspase 10* ^28^, downregulating *Bcl-XL* expression^36^ and increasing PARP and caspase 3 cleavage via JNK and p38 signal pathway activation^22^. TOPK also confers resistance to TRAIL-induced apoptotic cell death^37^, which, along with IκBα phosphorylation and RelA nuclear translocation^38^, link TOPK activity to aberrant NF-κB signalling and cancer progression. An increase in polyploidy following doxorubicin treatment indicates that mitosis occurs aberrantly for TOPK overexpressing cells as a response to damaged DNA^39^. Indeed, TOPK knockdown or inhibition enhances radiation sensitivity in a tumour-specific manner by impeding resolution of DNA damage and increasing apoptotic cell death^40^. Taken together, increased TOPK expression in cancer cells promotes the activation of multiple signalling pathways involved in reinforcing the hallmarks of cancer. Thus TOPK represents a master regulatory kinase whose manipulation has the potential to disrupt pro-carcinogenic processes, support the efficacy of DNA damaging agents and to overcome chemoresistance during cancer treatment.

### Development of TOPK inhibitors

Three inhibitors have been developed to specifically target TOPK: HI-TOPK-032;^41^ OTS514/OTS964;^27^ and ADA-07^42^ (Table 3). The first of these, HI-TOPK-032, suppressed proliferation of colon cancer cells in vitro and partially suppressed colorectal and nasopharyngeal xenograft growth in vivo^22,43^. More recently, ADA-07 showed potential as a chemopreventive and therapeutic agent in the treatment of skin cancer. Inhibition of TOPK by topical application of ADA-07 suppressed development of solar ultraviolet-induced tumour development in mouse skin following exposure to solar irradiation^42^.

**Table 3:** Characterisation of TOPK inhibitors—preclinical studies

Inhibitor	Model	Sensitivity	Specificty	References
HI-TOPK-032	Kinase activity [% Inhibition]	TOPK (60%) [2 μM HI-TOPK-32]	Other kinase targets: MEK1 (20%), ERK1 (0%), JNK1 (0%), p38 (0%)	^41^
	Colon cancer cell lines nasopharyngeal carcinoma cell lines	0.5–5 μM, 10 μM	↓ Growth, ↓ Anchorage-independent colony formation	^22, 41^
		0.5–8 μM	↑ DNA fragmentation and cell death, ↑ Intracellular ROS	^43^
	s.c. xenograft	HCT-116 [10 mg/kg t.i.w. (25 days)] CNF-2 [5 mg/kg t.i.w. (14 days)] HT-29 [5 mg/kg t.i.w. (20 days)]	↓ Tumour growth, No weight loss ↓ Tumour growth, No weight loss Chemosensitisation (oxaliplatin)	^41^
ADA-07	Kinase assay NHDF, A431 & SCC12 JB6 P +	Inhibition [5-10 μM ADA-07] 0.625-10 μM 1.25-10 μM	Co-precipitation, no MEK1 inhibition ↓ Growth, ↓ Anchorage-dependent colony formation ↓ Transformation potential	^42^
	SUV-induced papilloma formation	[0.1 mg, 1 mg (topical) t.i.w. (28 weeks)]	↓ Tumorigenesis (early-stage), ↓ Papilloma formation (late-stage) No cytotoxicity	^42^
OTS514	Kinase activity [% Inhibition]	TOPK (84%) [0.2 μM OTS514]	Other kinase targets: cSrc (61%), FLT3 (44%), FYN (19%), LYN (28%), CDK2/Cyclin A (60%), BTK (12%), DAPK1 (29%), GSK3b (25%), IGF1R (28%), IRAK4 (21%), RET (13%), TAK1 (42%)	^27^
	SCLC cell lines, Kidney cancer cell lines, Ovarian cancer cell lines	IC_50_ values: DMS114 (1.3 nM), H69AR (7.3 nM), H446 (8.4 nM), H69 (0.4 nM), H82 (7.2 nM), H146 (39.3 nM), H524 (2.6 nM), H2171 (42.6 nM), DMS273 (4.1 nM), SBC-3 (2.0 nM), SBC-5 (3.7 nM), VMRC-RCW (19.9 nM), Caki-1 (27.8 nM), Caki-2 (20.1 nM), 769-P (20.7 nM), 786-O (44.1 nM), CaOV3 (3 nM), OVTOKO (46 nM).	↓ Growth, ↑ apoptotic cell death Sensitivity is relative to intrinsic TOPK expression ↓ FOXM1 transcription ↓ TOPK protein expression	^8, 26, 44^
	s.c. xenograft	A549, LU-99 [1, 2.5, 5 mg/kg t.i.w. (14 days)]	↓ Tumour growth, Haematopoietic toxicity (free); slight reduction in body weight (liposomal, high dose)	^27^
	Metastatic growth	[25 mg/kg, q.d. (14 days) 50 mg/kg, q.d. (11 days)]	Abolition of tumour growth in 15% > 20% weight loss in high dose group ↓ Growth, Sensitivity does not vary according to tumour site or histological type	^44^
	Ex vivo (patient derived)	Ovarian cancer (10 nM, 100 nM)		
OTS964	Kinase activity [% Inhibition]	TOPK (80%) [2 μM OTS964]	Other kinase targets: cSrc (88%), FLT3 (72%), FYN (63%), LYN (77%), MELK (61%), CDK2/Cyclin A (44%), BTK (52%), DAPK1 (42%), GSK3b (45%), IGF1R (40%), IRAK4 (43%), PIM1 (59%), RET (41%), TAK1 (44%)	^27^
	Various cancer cell lines	IC_50_ values: A549 (31 nM), LU99 (7.6 nM), DU4475 (53 nM), MDA-MB-231 (73 nM), T47D (72 nM), Daudi (25 nM), UM-UC-3 (32 nM), HCT-116 (33 nM), HT29 (290 nM), MKN1 (38 nM), MKN45 (39 nM), HepG2 (19 nM), MIAPaca-2 (30 nM), 22Rv1 (50 nM), CaOV3 (14 nM), RMG-1 (110 nM), Hela (60 nM), MRC5 (185 nM), HFL-1 (175 nM), DU145 (43 nM), PC3 (147 nM), H1299 (88 nM), T24 (153 nM), SQ20B (59 nM), HAP1 (83 nM).	↓ Growth, ↑ apoptotic cell death Sensitivity is tumour cell line-specific, tumour-specific radiosensitisation	^27, 40^
	s.c. xenograft	LU-99 [40 mg/kg i.v. (×6 in 18 days) 50 or 100 mg/kg p.o.]	↓ Tumour growth with complete regression, transient haematopoietic toxicity	^27^

Key: *ROS* reactive oxygen species, *s.c.* subcutaneous, *i.v.* intravenous, *t.i.w.* three times per week, *SUV* solar ultraviolet radiation, *SCLC* small cell lung cancer, *q.d.* daily, *p.o.* oral dosing

Two novel high potency compounds (OTS514 and its methylated derivative, OTS964) effectively inhibit lung tumour growth in murine xenografts when delivered by either oral or intravenous routes^27^. TOPK inhibition with OTS964 or OTS514 abolished ex vivo growth of patient-derived ovarian cancer cells in a dose-dependent manner, and completely suppressed dissemination of ovarian malignancy in peritoneal xenografts^44^, although free OTS964 and OTS514 triggered significant weight loss and haematological toxicity during administration. Liposomal delivery was then adopted to improve tumour-specific uptake through the enhanced permeation and retention effect. This strategy prevented anaemia and leukocytopenia associated with intravenous treatment from developing, and enabled full recovery following oral delivery of the compounds. More recently, ^18^F-labelled OTS964 has shown favourable pharmacokinetics and biodistribution when injected intravenously in a glioblastoma-carrying mouse model^45^. Preclinical validation of [^18^F]FE-OTS964 is a promising first step towards the future use of TOPK inhibitors in a clinical context by enabling PET imaging of TOPK positive tumours.

Investigations into the mechanistic potential for tumour growth suppression by OTS514 in TOPK-expressing tumour cell lines have found that dose-dependent growth suppression and increased apoptotic cell death is associated with a reduction in FOXM1 activity and down-regulation of TOPK and MELK expression^8,26^. These studies hint at a role for TOPK in stem cell differentiation through regulation of FOXM1-mediated transcriptional activity and MELK signalling. Indeed, whilst in vivo administration of OTS514 causes leukocytopenia, it concurrently increases the megakaryocyte subpopulation and peripheral platelets in treated mice, indicating that differentiation of haematopoietic stem cells is altered by TOPK inhibition^27^. Furthermore, in vitro exposure to OTS964 decreases the CSC subpopulation in CD56 positive small cell lung cancer cell lines^26^.

### Conclusions and future directions

Current therapeutic strategies to interfere with the proliferative potential of cancer involve either inhibition of mitogenic activating factors, or manipulation of cell cycle regulatory proteins. Of the mitogenic activators, dual specificity MEK homologues are highly prised potential targets for tackling cancers with dysregulated EGFR, Ras or Raf activity^46,47^. The small molecule inhibitors of MEK1/2 developed to date are highly potent and selective for the MEK-ERK pathway, however, systemic toxicity and acquired resistance have been limiting factors in clinical development^48^. The most promising regulatory targets are cyclin-dependent kinases (CDKs) (most recently CDK4/6 selective inhibitors) and checkpoint kinases (particularly WEE1, CHK1 and PLK inhibitors). However, despite demonstrating high potency in preclinical studies, early clinical trials with first and second-generation pan-CDK inhibitors have been disappointing. More highly selective CDK4/6 inhibitors have produced more encouraging outcomes, although dosing is limited by neutropoenia, and efficacy is modest when these inhibitors are used as single agents^49,50^. It appears that in order to maximise cancer treatment strategies in the future, a range of potential options will be required in the clinician’s arsenal to personalise combination therapies and to combat refractory resistance. As an emerging alternative to the current cell cycle regulatory targets described above, TOPK’s chemotherapeutic potential is threefold:

1. TOPK expression is enhanced in tumours arising from numerous histological subtypes and is suppressed in non-transformed cells from differentiated tissues. Dysregulation of TOPK appears to promote tumour growth and progression by enabling cancer cells to overcome cell death signalling pathways, bypass regulatory checkpoint control mechanisms and migrate beyond their point of origin. Hence, inhibition of TOPK is an attractive biochemical strategy for overcoming tumour aggression, metastatic growth and therapeutic resistance.
2. Expression in untransformed tissues is relatively low and appears to be non-essential to development or cellular function. TOPK activity rises during the progression towards mitosis and peaks in prophase, however evidence suggests that it is functionally redundant for cell division. Cells with TOPK knockdown continue to undergo growth and proliferation in vitro, and *TOPK* knockout mice are healthy, unlike *MEK1*^*−/−*^ mice, which are non-viable^51^. Consequently, TOPK inhibition is less likely to be associated with normal tissue toxicity compared to existing MEK1/2 and CDK inhibitors.
3. TOPK facilitates the fidelity and duration of mitosis in actively dividing tissues, predominantly via its influence over checkpoint control proteins. Enhanced expression of TOPK supports the demands of increased proliferative activity in carcinogenic tissue by optimising mitotic efficiency. Equally, suppression of TOPK activity weakens the regulatory efficacy of mid-mitotic signalling, with the potential to elongate the transition between prophase and anaphase and either stall, or delay, mitotic progression. Mitotic defects facilitate tumorigenesis^52^, however, complete disabling of mitotic checkpoint control has been proposed as a potential anticancer strategy^53–55^.

Adaptation to TOPK-mediated signalling by cancer cells creates a dependence on TOPK for mitotic checkpoint control which can be exploited by chemotherapeutic inhibition in combination with DNA damaging agents. As yet, development of pharmaceutical strategies for targeting TOPK overexpression in cancer patients remain in the preclinical stage, and sensitivity and specificity for the majority of compounds developed to date is low. Of the possibilities outlined in this review, OTS514 and OTS964 are the only compounds which inhibit TOPK activity with high potency, however, these agents are particularly non-specific, with co-inhibition of other kinases demonstrated to various degrees in either case^27^. Both of these compounds have been associated with haematological toxicity in vivo, and therefore it is highly likely that their mechanism of action involves indirect targeting of multiple kinases, so their effect on tumour growth may be combinatorial. Nonetheless, novel MEK family members, such as TOPK, represent druggable targets of dysregulated signalling pathways with the potential to bypass the adverse outcomes posed by MEK1/2-targeting inhibitors. As single agents or in combination with therapeutics, selective TOPK inhibitors have the capacity to potentiate the impact of DNA damaging agents by abrogating cell cycle checkpoint control and by re-establishing pro-apoptotic signalling in resistant tumours.
